# CIZ1 in Xist seeded assemblies at the inactive X chromosome

**DOI:** 10.3389/fcell.2023.1296600

**Published:** 2023-12-13

**Authors:** Sajad Sofi, Dawn Coverley

**Affiliations:** ^1^ Department of Biology, University of York, York, United Kingdom; ^2^ York Biomedical Research Institute, University of York, York, United Kingdom

**Keywords:** X inactivation, *Xist* (X-inactive specific transcript), CIZ1, phase separation, nuclear matrix

## Abstract

There is growing evidence that X-chromosome inactivation is driven by phase-separated supramolecular assemblies. However, among the many proteins recruited to the inactive X chromosome by *Xist* long non-coding RNA, so far only a minority (CIZ1, CELF1, SPEN, TDP-43, MATR3, PTBP1, PCGF5) have been shown to form *Xist*-seeded protein assemblies, and of these most have not been analyzed in detail. With focus on CIZ1, here we describe 1) the contribution of intrinsically disordered regions in RNA-dependent protein assembly formation at the inactive X chromosome, and 2) enrichment, distribution, and function of proteins within *Xist*-seeded assemblies.

## Introduction

Transcriptional silencing of one copy of the X chromosome (X chromosome inactivation, XCI) occurs during early embryogenesis in female mammals (Lyon, 1961), and is the most extensively studied model of stably repressed chromatin formation. The initiating molecule is the 17-kb long noncoding RNA (lncRNA) X-inactive specific transcript (*Xist)* (Brockdorff et al., 1992; Brown et al., 1992), whose expression is restricted to one of the X chromosomes in female cells by the cis-acting activity of the antisense Tsix lncRNA (Lee et al., 1999), which suppresses *Xist*. The X chromosome that continues to express *Xist* generates approximately 100 molecules per cell, which initiate recruitment of chromatin modifying proteins across the inactive X chromosome (Xi), through interactions mediated by a series of repeat elements. Since its discovery more than 30 years ago, detailed analysis has revealed important insights into how this drives silencing of most of the genes on the Xi (Markaki et al., 2021; Rodermund et al., 2021), and has been reviewed extensively elsewhere including (Sahakyan et al., 2018; Brockdorff, 2019; Monfort and Wutz, 2020; Strehle and Guttman, 2020; Loda et al., 2022).

Recent studies have highlighted phase separation as part of the process underpinning Xi chromatin condensation and gene silencing (Cerase review), though its putative influence is far from established (Collombet et al., 2023). Phase separation is a process in which molecules spontaneously separate into a molecule-rich phase, which coexists in a cell with molecule-lean phase. The physical properties of size, shape, composition and behaviour of *Xist* nuclear foci is similar to other phase separated molecular condensates. Some *Xist* binding proteins (CELF1, SPEN, MATR3, TDP-43, PTBP1, PCGF5, and also CIZ1) have been shown to form *Xist*-seeded protein assemblies (Ridings-Figueroa et al., 2017; Pandya-Jones et al., 2020; Markaki et al., 2021; Rodermund et al., 2021; Jachowicz et al., 2022; Sofi et al., 2022), but the molecular mechanisms by which they arise remain largely unknown. In this review we discuss the role of intrinsically disordered prion-like domains (PLDs) in building RNA-protein assemblies at the Xi (Table 1), with focus on CIZ1 and its relationship with the repeat E element of *Xist*.

**TABLE 1:** Repeat E proteins known to form assemblies. Protein disorder predicted by PONDR (Mészáros et al., 2019) and MobDB-lite (Romero et al., 1997). n.p. means disorder for this protein is not predicted. PONDR score is indicative of the degree of disorder among residues or regions in a protein, not in an entire protein. PONDR scores greater than 0.5 suggest disorder. We express PONDR output as overall percent disorder in proteins. MobiDB lite does not give any score but generates overall percent disorder.

Name	Uniprot	PONDR disorder	MobiDB-LITE disorder
CIZ1	Q8VEH2	69.38%	50.4%
SPEN	Q96T58	74.13%	57.3%
PTBP1	P26599	37.52%	n.p.
CELF1	Q92879	50.21%	6.8%
MATR3	P43243	54.90%	36.5%

### Intrinsically disordered domains of CIZ1

CIZ1 is a ubiquitous nuclear protein that was identified through a yeast two-hybrid screen as an interaction partner of the cell cycle regulator p21/Cip1/CDKN1A, and later as a functional interactor of cyclin A-CDK2 (Mitsui et al., 1999; Coverley et al., 2005). It is linked with initiation of DNA replication (Copeland et al., 2010; Copeland et al., 2015), and because it remains in the nucleus after removal of chromatin is classified as a nuclear matrix protein (Ainscough et al., 2007). Its interaction with *Xist* emerged more recently, and has been shown to require the repeat E region of *Xist* (Chu et al., 2015; Ridings-Figueroa et al., 2017; Sunwoo et al., 2017; Dixon-McDougall and Brown, 2022). The biological significance of CIZ1’s function is apparent in a CIZ1 null murine model which develops female specific lymphoproliferative disorder (Brockdorff, 2019), and in the many human cancers of both sexes in which CIZ1 is dysregulated.

Though not required to establish XCI in the embryo, or to maintain global Xi silencing once established, in somatic cells (fibroblasts, B and T lymphocytes) CIZ1 is required to trap *Xist* transcripts at their source (Ridings-Figueroa et al., 2017). It is possible that it plays a similar role at other less visible loci because CIZ1 loss leads to failure to maintain tight control over genes under the regulation of the polycomb complex across the nucleus (Stewart et al., 2019). At the Xi, CIZ1 normally forms strongly enriched RNA-protein assemblies in a manner dependent on its two intrinsically disordered prion-like domains (PLD1 and PLD2) (Sofi et al., 2022). Both are alternatively-spliced, and excluded from some forms of CIZ1 in developmental and disease states (Warder and Keherly, 2003; Coverley et al., 2005; Dahmcke et al., 2008). Intrinsically disordered regions (IDRs) are amino-acid sequences with low sequence complexity and no fixed conformation (Alberti, 2017). CIZ1’s PLD1 is made up of nine blocks of 2-6 residue long polar uncharged glutamine repeats, interspersed with non-polar leucine or isoleucine residues. While PLD2 harbors no such repeats, it is made up of 15 (38.4%) glutamine residues interspersed with other polar amino acids. Approximately 5% of PLD2 residues are acidic, compared to 13% of non-PLD CIZ1, rendering it less negatively charged. Together, PLD1 and PLD2 support formation of concentration- and time-dependent CIZ1 assemblies possibly through multivalent weak interactions (electrostatic, cation-π, π-π stacking interactions), including dipole-dipole interaction of glutamine residues with aromatic groups. Collectively these interactions may favor CIZ1 self-assembly, as has been proposed for other poly Q proteins (Brangwynne et al., 2015; Protter et al., 2018).

**FIGURE 1:**
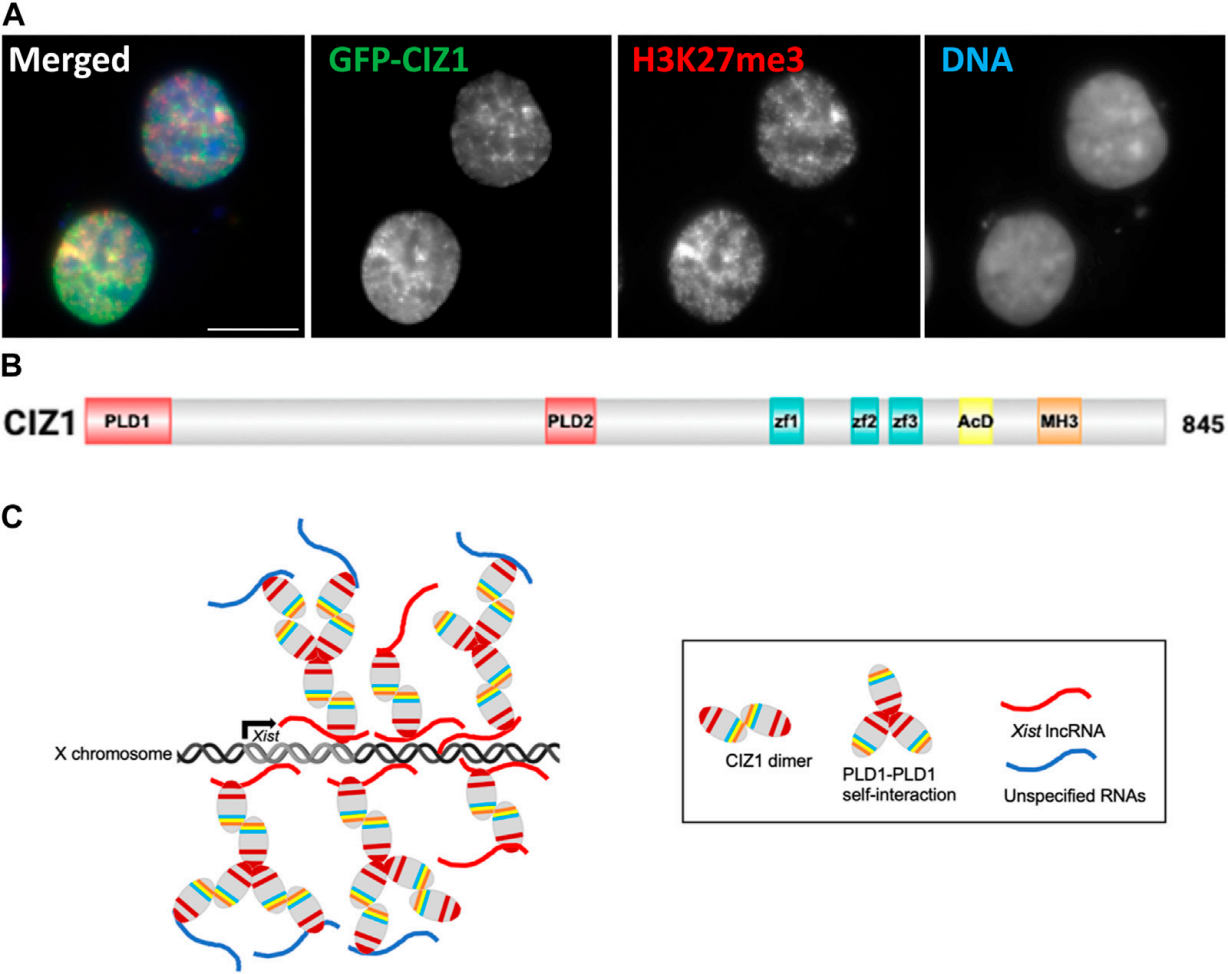
Formation of CIZ1 assemblies drive acquisition of modifications in CIZ1-null cells. **(A)** Image showing ectopic GFP-CIZ1 which drives acquisition of repressive histone post-translational modifications in CIZ1-null primary embryonic fibroblast cells (Sofi et al., 2022). Scale bar is 10 μm. **(B)** Summary of CIZ1 protein domains. PLD1 and PLD2 are red, Zf1-3 (zinc finger domains 1, 2 and 3 are blue; acidic domain (AcD) is yellow; and Matrin 3 domain (MH3) is orange. **(C)** Model summarising the emerging interpretation of information on CIZ1 in *Xist* seeded assemblies at Xi.

Purified PLD-containing CIZ1 fragments can alone form assemblies *in vitro,* but these are much larger (6 μm) in size than those observed inside the cell nucleus and resemble branched filaments rather than globular condensates. Moreover the same fragments are not sufficient to form assemblies at Xi in cultured cells, which requires additional functional domains (Sofi et al., 2022). Similar behavior has been observed for Whi3 RNA binding protein (Zhang et al., 2015), though the cellular mechanisms that control its assembly size and shape also remain poorly understood. For both proteins extrapolation from *in vitro* protein characterization to a cellular context is complicated by the buffering capacity of RNA (and other cellular factors), and the potential of specific RNA molecules to bridge protein interactions. In the case of CIZ1, this may influence both the extent of its self-interaction and also determine where in the nucleus assemblies are supported. Despite these uncertainties, analysis *in vitro* showed convincingly the contributions of the IDRs PLD1 and PLD2 to the formation of self-assemblies (Figure 1).

The majority (63%) of proteins contain IDRs (Tsang et al., 2020), which participate in nearly half of the total RNA-protein interactions in cells (Castello et al., 2016), and in some cases IDRs have been shown to modulate chromatin repression and gene silencing (Jachowicz et al., 2022). IDRs can bind RNA, DNA, proteins, and as purified proteins also phase separate *in vitro* (Protter et al., 2018, Brodsky et al., 2020)*.* While IDRs in some DNA-binding proteins bind specific sequences *in vivo* (Brodsky et al., 2020; Wang et al., 2023), the IDRs of RNA binding proteins, including CIZ1, appear to bind RNAs promiscuously, in some cases without any apparent sequence specificity (Protter et al., 2018; Sofi et al., 2022; Cubuk et al., 2023). The lack of RNA sequence specificity is attributed to their conformational flexibility (Varadi et al., 2015; Ottoz and Berchowitz, 2020) and overall charge, with positively charged IDRs binding to negatively charged RNAs (Protter et al., 2018). An unresolved question is whether IDRs contribute to functional RNA specificity inside cells. Some studies have suggested that repeat regions within some IDRs fold into secondary structures (α-helix, loops or random coil), which specifically interact with folded RNAs driving structural specificity (Protter et al., 2018; Zeke et al., 2022), possibly regulated by post-translational modifications (Ottoz and Berchowitz, 2020). Other studies propose that some structurally malleable RNAs have chaperone activity which could impose order upon IDRs, potentially augmenting the specificity of proteins towards RNA (Ottoz and Berchowitz, 2020; Luo et al., 2023). In the case of CIZ1’s IDRs no biophysical information on their relationship with RNA has yet been uncovered, but their contribution to stable interaction with RNA has been assessed (Sofi et al., 2022).

### Relationship between CIZ1 and *Xist* molecules

*Xist* lncRNA interacts with numerous proteins, some of which become enriched at Xi (Chu et al., 2015; McHugh et al., 2015; Moindrot et al., 2015; Monfort et al., 2015), and much effort is concentrated on understanding the molecular basis of their enrichment. *Xist* contains six differently sized repeat regions (A-F) implicated in recruitment of different *Xist* binding proteins (McHugh et al., 2015; Brockdorff, 2018; Brockdorff et al., 2020; Monfort and Wutz, 2020; Boeren and Gribnau, 2021; Raposo et al., 2021). Repeat E is comprised of two sets of tandem repeats (35 copies of a 16–27 bp C/U/G-rich element at the 5′ end, and 25 copies of a 6–19 bp C/U-rich element at the 3’ end) (Pandya-Jones et al., 2020; Brockdorff et al., 2020). It is largely unstructured and thought to act as a protein binding platform in cells (Smola et al., 2016). Its deletion from *Xist* blocks recruitment of CIZ1 to Xi in somatic cells, and CIZ1 binds directly to *Xist* repeat E *in vitro,* with a degree of sequence preference imparted by its PLDs (Ridings-Figueroa et al., 2017; Sunwoo et al., 2017; Sofi et al., 2022). *In vitro* experiments that compare affinity for repeat E over repeat A, absence of PLD1 dampened the former more than the latter, while absence of PLD2 relieved apparent suppression of interaction with repeat A, rendering the affinity for both RNA elements similar (Sofi et al., 2022). This highlights a complex relationship with respect to specificity, and implies that PLD2 may enhance the affinity of PLD1 for repeat E by dampening affinity for other (unknown) RNAs. Moreover, removal of either of the IDRs (PLD1/PLD2) from CIZ1 causes failure to concentrate *Xist* transcripts at Xi in differentiated fibroblasts (Sofi et al., 2022), which further suggests that the interplay between them is important to specify interaction with *Xist* and thus the location at which CIZ1 assemblies form *in vivo*. In contrast, *Xist* dispersal is not observed upon deletion of the IDR in SPEN (Jachowicz et al., 2022). A structural examination of the relationship between PLD1 and PLD2, in complex with structurally flexible *Xist,* is now needed in order to understand how they cooperate to drive CIZ1 assembly formation at Xi.

There is consensus now that approximately 100 *Xist* RNA molecules are present in female cells, and are confined to ∼50 *Xist* foci, each containing a pair of *Xist* molecules (Markaki et al., 2021; Rodermund et al., 2021). Surprisingly, the number of *Xist* foci increased in differentiating embryonic stem cells (ESCs) when repeat E was deleted, which implicates repeat E in the integrity or stability of *Xist* pairs (Pandya-Jones et al., 2020). In experiments where CIZ1 is depleted, *Xist* pairs remain largely intact arguing against a role for CIZ1 (Rodermund et al., 2021), even though it is itself a homodimeric entity (Turvey et al., 2023). In fact, the bridging entity remains enigmatic and could depend on RNA-RNA interactions (Van Treeck et al., 2018) as on its own, repeat E forms micrometer sized droplets (Pandya-Jones et al., 2020; Ma et al., 2021).

Recently expression of Halo-tagged transgenes in Xist^MS2−GFP^ cells followed by imaging with 3D-SIM, revealed 1:1 binding stoichiometry between CIZ1 and *Xist*, and similar stoichiometry for other repeat E binding proteins (CELF1, PTBP1, MATR3, TDP-43) (Pandya-Jones et al., 2020; Markaki et al., 2021). However, unlike these other factors the strong enrichment of endogenous CIZ1 around the Xi, detected by immunofluorecence microscopy, appears to be inconsistent with 1:1 stoichiometry across the whole assembly. As mixed RNA can drive enlargement of CIZ1 networks *in vitro* and CIZ1 also has a propensity for self-interaction (Sofi et al., 2022) we hypothesise that, while *Xist* might specify where CIZ1 assemblies form in the nucleus, their enlargement in somatic cells is amplified independently of *Xist*. This idea is supported by a recent study which shows that SPEN, which directly binds *Xist* repeat A, amplifies its abundance by forming assemblies with other SPEN molecules driven by multivalent interactions between their IDRs (Jachowicz et al., 2022).

### Spatial distribution of CIZ1 within *Xist*-nucleated assemblies

Although CIZ1 is normally recruited during the initiation phase of XCI it is not required at this critical point in development, and CIZ1-null mice develop normally (Ridings-Figueroa et al., 2017). A reliance on CIZ1 for *Xist* retention becomes evident in the later maintenance phase of XCI (Stewart et al., 2019), which is consistent with a delayed requirement for the repeat E element (Pandya-Jones et al., 2020). Despite this, focus has fallen on the precise order in which CIZ1 and other repeat E binding proteins are recruited to the pre-Xi during the initiation phase (Pandya-Jones et al., 2020; Markaki et al., 2021). It has been postulated that CIZ1 forms a stable ‘core’ of the supramolecular protein complexes (SMACs) that are nucleated by *Xist*, because its dwell time at Xi, determined by fluorescence recovery after photobleaching (FRAP) is by far the highest among the known *Xist* repeat E proteins (Table 2) (Markaki et al., 2021). In the same study CIZ1 concentration in *Xist-*SMACs did not fluctuate and remained constant, unlike SPEN and CELF1 whose concentration gradually increased (Pandya-Jones et al., 2020; Markaki et al., 2021). However, questions remain about the nature and purpose of a putative ‘core’ structure. CIZ1’s repeat E-driven recruitment does not appear to increase the local concentration of other repeat E-dependent protein factors, as no CIZ1-dependent enrichment was found in ES cells, even though it interacts with MATR3 and PTBP1 in co-immunoprecipitation experiments (Strehle and Guttman, 2020). Similarly in our unpublished observations CIZ1 does not enrich SAF-A, PTBP1 or MATR3 in female fibroblasts, rather we noticed an under representation of these proteins at sites of *de novo* CIZ1 assemblies. Furthermore, CELF1, PTBP1, TDP-43 and MATR3 interact with each other to form a heteromeric protein assembly on repeat E and this assembly does not contain CIZ1 (Pandya-Jones et al., 2020), which suggests that distinct protein assemblies could form on repeat E, possibly in dynamic equilibrium with each other. Therefore, from the available data it is not clear that CIZ1 forms a core, at least not one with a positive influence on other factors. Conversely, limited data suggest that CIZ1 may form a protective ‘shell’ or molecular shield (Mészáros et al., 2019) around the Xi that excludes or includes soluble factors. When CIZ1-Xi assemblies are destabilized by the over-expression of interfering fragments (Turvey et al., 2023), or absent as in CIZ1-null cells (Stewart et al., 2019), underlying chromatin becomes depleted of PRC1-dependent ubiquitination of H2AK119. This might be driven by inappropriate exposure of chromatin to deubiquitylating enzymes (DUBs) is suggested because DUB inhibition abrogates the loss of H2AK119ub in both experimental contexts (Turvey et al., 2023). These data are beginning to argue that the CIZ1 in *Xist-*nucleated assemblies becomes essential only in the later stages of XCI because its primary role is to protect the status of chromatin that was established earlier.

**TABLE 2:** Dwell time of repeat E proteins at the Xi and in the nucleoplasm (Markaki et al., 2021).

Protein	Nuclear (min/secs)	Xi (min)
CIZ1	16.5	19.1
CELF1	56	1.7
PTBP1	1.4	1.8
SPEN	1.1	2.4
